# Cloning and characterization of a novel sigma-like glutathione S-transferase from the giant panda parasitic nematode, *Baylisascaris schroederi*

**DOI:** 10.1186/s13071-014-0629-9

**Published:** 2015-01-23

**Authors:** Yue Xie, Xuan Zhou, Lin Chen, Zhihe Zhang, Chengdong Wang, Xiaobin Gu, Tao Wang, Xuerong Peng, Guangyou Yang

**Affiliations:** Department of Parasitology, College of Veterinary Medicine, Sichuan Agricultural University, Ya’an, 625014 China; Centre for Animal Diseases Control and Prevention, Dachuan Animal Husbandry Bureau, Dazhou, 623000 China; Chengdu Research Base of Giant Panda Breeding, Chengdu, 610081 China; China Conservation and Research Center for Giant Panda, Wolong, 623006 China; Department of Chemistry, College of Life and Basic Science, Sichuan Agricultural University, Ya’an, 625014 China

**Keywords:** *Baylisascaris schroederi*, Giant panda, Glutathione S-transferase, Sigma class, *Bsc*-GSTσ, Immunolocalization, Immunodiagnosis

## Abstract

**Background:**

*Baylisascaris schroederi*, an intestinal nematode of the giant panda, is the cause of the often fatal disease, baylisascariasis. Glutathione S-transferases (GSTs) are versatile enzymes that can affect parasite survival and parasite-host interactions and, are therefore, potential targets for the development of diagnostic tests and vaccines.

**Methods:**

In this study, we identified a full-length cDNA that encoded a novel, secretory sigma-like GST (*Bsc*-GSTσ) from a *B. schroederi*-omic dataset. Following cloning and sequencing, sequence and structural analyses and comparative modeling were performed using online-bioinformatics and proteomics tools. The recombinant *Bsc*-GSTσ (r*Bsc*-GSTσ) protein was prokaryotically expressed and then used to detect antigenicity and reactivity using immunoblotting assays. In addition, the native protein in female adult *B. schroederi* was located via immunofluorescence techniques, while the preliminary ELISA-based serodiagnostic potential of r*Bsc*-GSTσ was assessed in native and infected mouse sera.

**Results:**

*Bsc*-GSTσ contained a 621-bp open reading frame that encoded a polypeptide of 206 amino acids with two typical sigma GST domain profiles, including a GST_N_Sigma_like at the N-terminus and a GST_C_Sigma_like at the C-terminus. The presence of an N-terminal signal sequence indicated that *Bsc*-GSTσ was a secretory protein. Sequence alignment and phylogenetic analyses showed that *Bsc*-GSTσ was a nematode-specific member of the Sigma class GSTs and shared the closest genetic distance with its homologue in *Ascaris suum*. Further comparative structure analyses indicated that *Bsc*-GSTσ possessed the essential structural motifs (e.g., βαβαββα) and the consensus secondary or tertiary structure that is typical for other characterized GSTσs. Immunolocalization revealed strong distributions of native *Bsc*-GSTσ in the body hypodermis, lateral chords, gut epithelium, gut microvilli, oviduct epithelium, and ovaries of adult female worms, similar to its homologue in *A. suum*. Building on good immunogenic properties, r*Bsc*-GSTσ-based ELISA exhibited a sensitivity of 79.1% and a specificity of 82.0% to detect anti-*B. schroederi* IgG antibodies in the sera of experimentally infected mice.

**Conclusion:**

This study presents a comprehensive demonstration of sequence and structural-based analysis of a new, secretory sigma-like GST from a nematode, and its good serodiagnostic performance suggests that r*Bsc*-GSTσ has the potential to detect *B. schroederi* and, therefore, could be used to develop an ELISA-based serological test to diagnose baylisascariasis in giant pandas.

## Background

The giant panda (*Ailuropoda melanoleuca*) is one of the world’s most iconic and endangered species, and is currently confined to several small mountain habitats of western China (Qinling, Minshan, Qionglai, Daxiangling, Xiaoxiangling, and Liangshan) with a population size of ~1600 [1,2]. Wild giant pandas face the threat of extinction from human population expansion, destruction of their habitat, and the detrimental impacts of parasites and other diseases [3,4]. Baylisascariasis, caused by the nematode, *Baylisascaris schroederi* (Nematoda: Ascaridida), is one of the leading causes of death for both wild and captive giant pandas and has been responsible for half of the recorded deaths of pandas from 2001 to 2005 [3,5]. As with other ascaridoids, adult *B. schroederi* usually inhabit the intestines of the giant panda, while the larvae may disseminate into various body tissues. In pandas, damage to bodily tissues includes extensive inflammation and scarring of the intestinal wall and parenchyma of the liver and lung (also known as visceral larval migran, VLM; caused by larvae), as well as intestinal obstruction, inflammation, and even death (caused by adults) [5-8]. Currently, diagnosis and identification of *B. schroederi* infection in pandas relies on morphological examination of fecal eggs, which requires extensive expertise and is difficult, laborious, and prone to error (as the density of eggs in bamboos-enriched feces is low and subject to possible environmental cross-contaminating with the eggs of other parasites, including morphologically similar *Baylisascaris* spp. [5]). Recently, a new molecular method to detect *B. schroederi* was developed based on the PCR-based detection of mitochondrial makers (COII or 12S) [9-11]. This method, however, cannot diagnose migrating larvae or adults outside of the egg-laying period. Hence, an alternative and more efficient molecular tool is needed. Serodiagnosis, particularly the ELISA tests (enzyme-linked immunosorbent assays) equipped with target molecules that play excretory/secretory (ES) roles and function in the survival, development, and immune evasion of parasites [12], would be an ideal and better strategy due to its sensitivity and clinical practices.

Glutathione S-transferases (GSTs; EC 2.5.1.18) are a versatile protein superfamily that are widely distributed among all living cells and act in cellular detoxification and protection via either catalyzing toxin conjugation with reduced glutathione (GSH) or passively binding to various exogenous/endogenous toxic molecules, including carcinogens, therapeutic agents, and products of oxidative stress [13,14]. For parasites, some secretory GSTs are further believed to be associated with parasite survival, repair of damage caused by host’s immune-initiated reactive oxygen species (ROS), transportation or metabolism of essential materials, and host immune modulation [12,14-19]. Encouragingly, because of these important functions, some parasite-derived GSTs, including those of parasitic nematodes, have been selectively targeted for vaccine development and diagnosis purposes [15,16,19-23]. For example, a secretory sigma-class GST from *Ascaris lumbricoides* (GSTA) has recently been identified and investigated as a new allergen for clinical diagnosis of the human roundworm disease [24], although the frequency of the antibody (mainly IgE) sensitization to GSTA is not high and the GSTA exhibits several isoforms with differential IgE recognition. Also, another secretory GST-3 from the human filarial nematode *Onchocerca volvulus* (OvGST3) is under investigation as a potential antigen candidate for the diagnosis of onchocerciasis due to its high exposure to the human host’s immune system and good immunogenic properties [19].

Given that most recently described nematode-derived GSTs are from the Sigma class in term of their sequence homology, structure, substrate specificity, immunological and phylogenetic analyses [20,22], and that no information on GSTs of *B. schroederi* is available to date; importantly, *B. schroederi*-specific immunogenic proteins as potential diagnostic agents are lacking, herein, the aims of this study were to (i) clone and express a new, secretory sigma-like GST, *Bsc*-GSTσ, from *B. schroederi*; (ii) characterize its potential functions by locating the native protein in adult parasites; and (iii) test the immunogenicity and preliminary ELISA-based diagnostic potential of its recombinant *Bsc*-GST (r*Bsc*-GSTσ) in mice using native and infected sera. The results of this work will provide the foundation for the further development of *Bsc*-GST as a candidate serodiagnostic antigen to detect *B. schroederi* in the giant panda.

## Methods

### Ethics statement

This study was reviewed and approved by the Animal Ethics Committee of Sichuan Agricultural University (AECSCAU; Approval No. 2011–028). Animals were handled strictly accordance with the animal protection law of the People’s Republic of China (released on 09/18/2009) and the National Standards for Laboratory Animals in China (executed on 05/1/2002).

### Animals

Female specific-pathogen-free (SPF) BALB/c mice (6–8 weeks old) were purchased from the Laboratory Animal Center of Sichuan University (Chengdu, China). New Zealand white rabbits were obtained from the Laboratory Animal Center of Sichuan Agricultural University (Ya’an, China). All animals were housed under a barrier environment in sterile cages and provided with pelleted food and sterilized water *ad libitum*. Animals were acclimated to these conditions for one week prior to the experiment.

### Parasites

*B. schroederi* female adults derived from naturally infected giant pandas were provided by the Department of Parasitology, College of Veterinary Medicine, Sichuan Agricultural University. Adult female *Ascaris suum* and *Baylisascaris transfuga* were isolated from infected pigs at a local slaughterhouse in Ya’an and an infected polar bear after treatment with pyrantel pamoate in Chengdu zoological garden, China, respectively. Embryonated and/or un-embryonated eggs were obtained from the respective dissections of the uteruses of *B. schroederi*, *B. transfuga*, and *A. suum* using established procedures [25]. The infective egg-L2 larvae of these three ascaridoids were collected from subsequent incubation of the embryonated eggs according to the USEPA and Tulane methods [26,27]. All L2-contained eggs were stored at 4°C until use.

### RNA isolation, amplification and bioinformatic analysis of *Bsc*-GSTσ-1

Total RNA was isolated from an adult female specimen of *B. schroederi* using an RNA extraction kit (Clontech, Palo Alto, CA) according to the manufacturer’s directions. The isolated RNA was subsequently subjected to first-strand cDNA synthesis using a cDNA synthesis kit and an oligo (dT)_18_ primer (MBI Fermentas, Germany). The resulting double-stranded cDNA was used as the template for PCR amplification with the sense primer (5′-AAGCAACATGCCGCAGTACAAG-3′) and the antisense primer (5′-CACAAAAAACAGAATAGACCCTAATA-3′) designed to target a full-length coding sequence of the GST homologue that was screened from the assembled and annotated *B. schroederi* genome (Scaffold ID 47) and transcriptome (Unigene ID 86248) datasets (data unpublished). The amplified product was gel-purified, cloned into the pMD19-T vector (TaKaRa, Dalian, China) and then sequenced. After a homology search by BLASTn (http://blast.ncbi.nlm.nih.gov/Blast.cgi?PROGRAM=blastn&PAGE_TYPE=BlastSearch&LINK_LOC=blasthome), the cDNA showed similarity to known sigma GSTs and therefore was designated as *Bsc*-GSTσ. The open reading frame (ORF) and deduced amino acid sequence of *Bsc*-GSTσ were derived using an Open Reading Frame Finder and the Lasergene software package for Windows (DNASTAR, Madison, WI, USA) and then assessed with ExPASy online servers (http://www.expasy.org/). Conserved domains (CD) were identified using the CD-Database-based PROSITE profile analysis (http://www.ncbi.nlm.nih.gov/Structure/cdd/cdd.shtml). The isoelectric point (pI) and molecular weight (Mw) of *Bsc*-GSTσ were calculated using the Compute pI/Mw tool (http://web.expasy.org/compute_pi/) and the signal sequence was predicted with the SignalP 4.0 server (http://www.cbs.dtu.dk/services/SignalP/). Similarity comparisons with previously reported sequences were conducted using DNAMAN 3.0 (Lynnon Biosoft, Quebec, Canada) and online BLASTp tool (http://blast.ncbi.nlm.nih.gov/Blast.cgi?PROGRAM=blastp&PAGE_TYPE=BlastSearch&LINK_LOC=blasthome). On the basis of observed similarities, a multiple sequence alignment and phylogenetic analysis were conducted. Sequences were aligned with ClustalW2 and the phylogenetic tree was constructed using the neighbor-joining (NJ) method [28] and plotted with MEGA 5.0 [29]. In addition, for structural modeling of *Bsc*-GSTσ, we used the YASPIN secondary structure prediction program (http://www.ibi.vu.nl/programs/yaspinwww/) to infer secondary structure. Tertiary (3D) structure was assessed through the CPHmodels-3.2 Server online program (http://www.cbs.dtu.dk/services/CPHmodels/) and by referring to the 1.90 Å resolution crystal structure of *N. americanus* GSTσ2 (PDB accession no.: 2ON5).

### Expression and purification of r*Bsc*-GSTσ

Due to the presence of the predicted signal peptide, a region encoding mature *Bsc*-GSTσ was amplified by PCR for expression using the following primers: 5′-CCC*GGATCC*ATTCGTGGCCTGGGTG-3′ (forward; a *BamH*I site in italics) and 5′-CGC*AAGCTT*CACAAAAGCAGAAGAGACTCTAATA-3′ (reverse; a *Hind*III site in italics). After enzyme digestion with *BamH*I and *Hind*III (TaKaRa) and gel-purification, this fragment was ligated into the plasmid expression vector pET32a (+) (Novagen, Madison, USA). The correct resulting plasmid was confirmed by sequencing, transformed into *E. coli* BL21 (DE3) cells (Invitrogen, Carlsbad, USA), and then grown in Luria-Bertani (LB) broth with 100 mg/mL of ampicillin at 37°C until the optical density at 600 nm reached 0.6. Expression was induced by adding 1 mM isopropyl β-D-thiogalactopyranoside (IPTG) for an additional 4 h culture at 37°C. The cells were pelleted and suspended in lysis buffer [50 mM NaH_2_PO_4_ (pH 8.0), 10 mM Tris–HCl (pH 8.0), 100 mM NaCl]. The samples were subjected to sonication and centrifuging to enable collection of inclusion bodies and cellular debris and the removal of other soluble substances. His_6_-tagged recombinant *Bsc*-GSTσ proteins were expressed as inclusion bodies in the pellets after sodium dodecyl sulfate-polyacrylamide gel electrophoresis (SDS-PAGE) and then purified using Ni^2+^ affinity chromatography (Novagen) under denaturing conditions according to the manufacturer’s protocol. Refolding of the purified recombinant proteins was performed as recommended elsewhere [8]. Thereafter, the refolded protein was concentrated using the vacuum freeze-drying technique and its concentration was determined with the micro-bicinchoninic acid protein assay reagent (Pierce/Thermo Fisher Scientific, Asheville, USA). Potential contamination by endotoxins was assessed using the limulus amoebocyte lysate-based gel-clot assay [30].

### Sera

Mouse immune sera against parasites *A. suum* (15 samples), *B. transfuga* (15 samples) or *B. schroederi* (43 samples) were produced for serodiagnostic assays as previously described [8,31,32]. Rabbit anti-*B. schroederi* sera were generated as follows: 3,600 *B. schroederi* infective embryonated eggs were administered to one New Zealand white rabbit, followed by four repeat inoculations every 2 weeks. The rabbit was bled two weeks after the final inoculation and the serum was collected for immunoblotting analysis. To obtain mouse polyclonal sera against r*Bsc*-GSTσ, 10 BALB/c mice were subcutaneously immunized with 50 μg of purified r*Bsc*-GSTσ mixed with FCA (Sigma, St. Louis, USA), followed by two booster immunizations (two weeks apart) using the same route and dose in the same adjuvant. Mice were bled two weeks after the final immunization. The anti-r*Bsc*-GSTσ sera were mixed and stored at −20°C until use. Additionally, 20 mouse native sera were provided by the Department of Parasitology, College of Veterinary Medicine, Sichuan Agricultural University.

### Immunoblotting and immunolocalization

For immunoblotting analysis, r*Bsc*-GSTσ was lysed in an electrophoresis sample buffer, run on 10% SDS-PAGE and subsequently transferred onto the nitrocellulose membrane. The blotting membrane was incubated with 5% skim milk in Tris-buffered saline (TBS) buffer for 1 h. To better test the antigenicity of r*Bsc*-GSTσ, rabbit immune sera from animals repeatedly inoculated with *B. schroederi* embryonated infective eggs, anti-r*Bsc*-GSTσ mice serum, and native rabbit or mouse serum were included here. After the membrane was washed three times with TBS-Tween 20 (TBST), it was further incubated for 2 h with 1:200 diluted alkaline phosphatase-conjugated goat anti-mouse or anti-rabbit IgG (ICN Pharmaceuticals, Costa Mesa, CA). Following the same washing steps described above, the protein signals were visualized using nitroblue tetrazolium and 5-bromo-4-chloro-3-in-dolylphosphate (NBT/BCIP; USB, Cleveland, OH) and recorded using Image Quant LAS-4000 (GE Healthcare Life Sciences, USA). For immunolocalization studies, adult female *B. schroederi* sections were probed with specific mouse anti-r*Bsc*-GSTσ serum (1:100) and then with fluorescein isothiocyanate (FITC)-labeled goat anti-mouse IgG (1:100; Santa Cruz, CA, USA) as described elsewhere [33]. The stained samples were mounted in glycerol/phosphate buffer (v/v, 9:1) and viewed under an Olympus BX50 fluorescence microscope (Olympus, Japan).

### ELISA

To assess the preliminary serodiagnostic potential of r*Bsc*-GSTσ, *B. schroederi*-specific IgG antibodies of mice were detected by ELISA following the methodology described by Vlaminck et al. [34], with some modifications. In brief, ELISAs were performed in polystyrene 96-well microtiter plates (Invitrogen) using 100 μL reaction mixtures with r*Bsc*-GSTσ antigen coated at three different concentrations (1, 2, and 4 μg/mL) in 0.1 M carbonate buffer (pH 9.6). After overnight incubation at 4°C, all plates were washed with PBS + 0.5% Tween 20 (PBST20) and then the wells were blocked with 100 μL of PBS-2% bovine serum albumin (BSA) for 2 h at 37°C. A serial two-fold dilutions (100 μL; ranging from 1:100 to 1:1600) of the positive serum sample (provided by our lab) and goat anti-mouse IgG-HRP conjugate (100 μL; 1:500) (Boster Bio-project Co., Wuhan, China) were used in the following steps, and positive sera and conjugates were diluted in PBS. Antibody binding was detected with 100 μL of O-phenylenediamine dihydrochloride substrate (0.4 mg/mL OPD, 50 mM dibasic sodiumphosphate, 25 mM citric acid, and 30% H_2_O_2_), and the optical density (OD) was measured at 492 nm. Negative and blank controls were included on each plate. After the best dilutions of r*Bsc*-GSTσ antigen and mouse serum were determined, 93 mouse serum samples (43 for *B. schroederi*-positive, 30 for *A. suum*/*B. transfuga*-positive, and 20 for control) were serodiagnosed with the ELISA. The sensitivity of the assay was calculated as follows: sensitivity (%) = ELISA positive/true positive × 100, while the specificity was evaluated by the cross-reaction with the heterosera derived from mice infection with the congeneric species, *B. transfuga*, and the related ascaridoid, *A. suum*.

### Statistical analysis

ELISA data were expressed as the mean value ± standard deviation (SD). Comparisons between test sera groups were carried out using one-way ANOVA in SPSS version 17.0 for Windows (SPSS Inc., Chicago, IL). *P* values <0.05 were considered to be significant. The negative cut-off was calculated as the mean value + 3× SD from the OD values of the normal sera, as previously described [35]. Additionally, the nucleotide sequence determined in the present study was deposited in the GenBank database under accession number KM435257.

## Results

### Molecular characterization of *Bsc*-GSTσ

**Figure 1:**
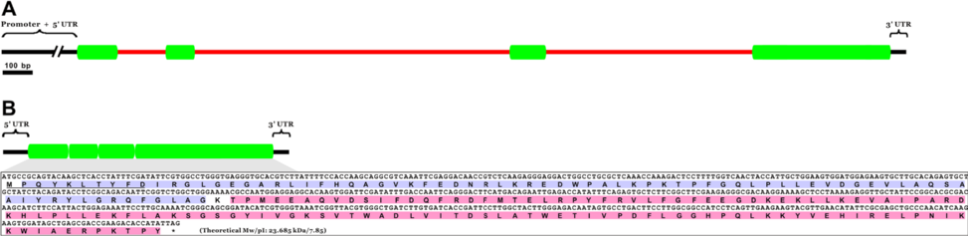
**Organization of genomic DNA and complementary DNA of** ***B. schroederi*** **sigma-like GST (** ***Bsc*** **-GSTσ). (A)** Schematic diagram showing the genomic organization of *Bsc*-GSTσ. Green blocks represent exons and the red lines between the exons represent introns. The promoter, 5′-UTR, and 3′-UTR regions are also denoted at both ends in black lines. **(B)** The full-length cDNA and its coding region of *Bsc*-GSTσ, spliced from **(A)**. The ORF and deduced amino acid sequences are detailed below the box. The frame stop codon is marked with an asterisk and the predicted signal peptide is underlined. The predicted N- and C-terminal GST domain profiles are shaded in light blue and pink, respectively. The predicted molecular weight (Mw) and isoelectric point (pI) are indicated at the end of the polypeptide.

The novel sigma-like GST, *Bsc*-GSTσ, was initially identified through homologously screening the assembled and annotated *B. schroederi* genome and transcriptome datasets. The full-length *Bsc*-GSTσ gene sequence within the genomic DNA was 4,340 bp in size and comprised four exons (95, 124, 133, and 269 bp) and three introns (160, 386, and 546 bp) (Figure 1A). The nucleotide sequence at the splice junctions is consistent with the canonical GT-AG rule [36]. The full-length cDNA of *Bsc*-GSTσ was 725 bp in size and contained a single ORF of 621 bp, a 5′untranslated region (UTR) of 30 bp, and a 3′ UTR of 74 bp (Figure 1B). The complete ORF of *Bsc*-GSTσ isolated here encoded a polypeptide of 206 amino acids with a predicted Mw of 23.685 kDa and a pI of 7.85. The PROSITE domain analysis revealed that the deduced *Bsc*-GSTσ polypeptide had two typical GST domain profiles at the N-(2–79 amino acids) and C-(81–206 amino acids) termini (see Figure 1B), which matched well with the coding domains of GST_N_Sigma_like (CDD accession no.: cd03039) and GST_C_Sigma_like (CDD accession no.: cd03192). In addition, the first ten amino acids corresponded to a signal peptide (Figure 1B), indicating that *Bsc*-GSTσ was a secretory GST. Removal of the signal peptide would result in a mature protein with a Mw of 22.398 kDa and a pI of 8.05. Four different *Bsc*-GSTσ clones were sequenced in this study, and no differences were found among them at the amino acid level.

**Figure 2:**
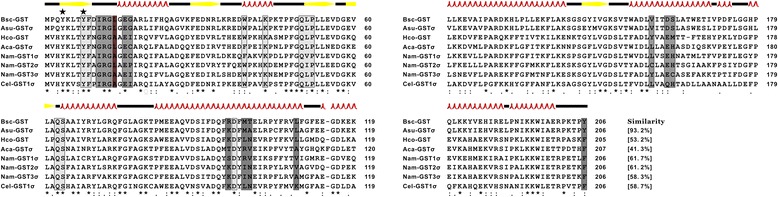
**Sequence alignment and secondary structure modeling of** ***Bsc*** **-GSTσ with nematode homologous GSTs.** For the alignment, the following sequences were retrieved from the NCBI protein database (species and accession numbers are indicated in parentheses) and aligned using the ClustalW2 program: *Bsc*-GSTσ (*B. schroederi*; KM435257), *Asu*-GSTσ (*A. suum*; P46436), *Hco*-GST (*H. contortus*; Q9NAW7), *Aca*-GSTσ (*A. caninum*; AAT37718), *Nam*-GST1σ (*N. americanus*; ACX53261), *Nam*-GST3σ (*N. americanus*; ACX53262), *Nam*-GST3σ (*N. americanus*; ACX53263), and *Cel*-GSTσ (*C. elegans*; P91253). Regions of identity (*), strong similarity (:) and weak similarity (.) are indicated. Gaps marked by hyphens are introduced for better alignment. Percentages of sequence similarity with respect to *Bsc*-GSTσ are shown at the C-terminus. The inferred binding sites for GSH (light gray), substrate (dark gray) or both (puce) [43] are indicated by boxes, and two catalytic Tyr residues are marked by black stars. For the secondary structure of *Bsc*-GSTσ, the elements including coils, strands, and helixes are shown above the alignment by black lines, yellow arrows, and red loops, respectively.

Multiple sequence alignment revealed that at the protein level *Bsc*-GSTσ shared the highest similarity (93.2%) with a GSTσ from *A. suum* (GenBank accession no: P46436), followed by three GSTσs from *N. americanus* (GenBank accession nos.: ACX53261-ACX53263, mean similarity of 60.4%) and GSTσ/GSTs from two parasitic nematodes *Ancylostoma caninum* (GenBank accession no.: AAT37718) and *Haemonchus contortus* (GenBank accession no.: Q9NAW7) as well as a free-living nematode *Caenorhabditis elegans* (GenBank accession no.: P91253) (41.3-58.7%; Figure 2). *Bsc*-GSTσ however showed a very low sequence similarity to GSTs from other organisms including flatworms (e.g., trematodes), insects (e.g., fruitflies), and other mammals (e.g., pandas and swine; data not shown). Further, the amino acid-based alignment demonstrated that the N-terminal region appeared to be highly conserved while the C-terminal region was diverse. For instance, several identical GSH-binding moieties (Tyr-8, Phe-9, Tpr-39, Lys-43, Pro-52, Gln-63, and Asp-97) in the N-terminal domain across all examined lineages were also present in the aforementioned nematode species and occurred without any substitutions in *B. schroederi* (Figure 2). More importantly, two tyrosine residues associated with the stabilization of GSH [14] were found to be conserved here (Tyr-4 and Tyr-8), including those in *Bsc*-GSTσ. Conversely, extensive variation was observed at the putative substrate binding pocket (H-site) located at the C-terminal domain, consistent with the diversified substrate specificities of members of the enzyme family toward xenobiotics. The corresponding substrate-binding moieties in *Bsc*-GSTσ were inferred and included: Ile-11, Arg-12, Gly-13, Glu-16, Gly-17, Arg-96, Met-99, Thr-100, Leu-109, Val-159, Asp-162, Ser-163, and Tyr-206 (dark gray boxes in Figure 2).

### Annotation of *Bsc*-GSTσ structural model

**Figure 3:**
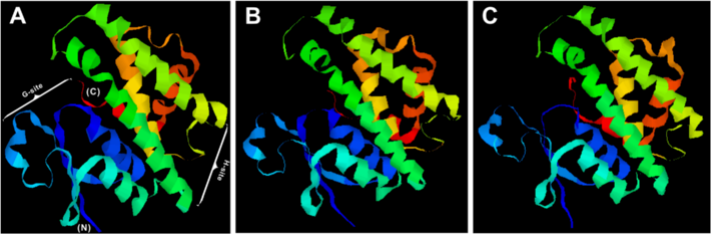
**3D models of (A)** ***Bsc*** **-GSTσ and two homologous proteins from (B)** ***A. suum*** **and (C)** ***C. elegans*** **.** The 3D structures were modeled by referring to the crystal structure of *N. americanus* GSTσ2 (PDB accession no.: 2ON5) using the CPHmodels-3.2 Server program (http://www.cbs.dtu.dk/services/CPHmodels/). N-terminal domain (βαβαββα motif) constituting G-site and C-terminal domain (α helix motif) constituting H-site are shown in **(A)**.

Similar to other homologous GSTs, the secondary structure of the *Bsc*-GSTσ protein consisted of a conserved βαβαββα motif in the N terminus (ranging from 3 to 74 residues) and a near complete α helix motif (ranging from 81 to 200 residues) in the C-terminus (Figure 2). Based on this secondary structure and the X-ray structure of *N. americanus* GSTσ2 (PDB no.: 2ON5), the 3D structure of *Bsc*-GSTσ was established and shown in Figure 3A. Interestingly, a G-site (GSH interactive region) built by the βαβαββα motif and an H-site built by the near complete α helix motif were determined in the N- and C-terminal domains, respectively, which was consistent with that of other nematode GSTs characterized to date, including *A .suum* (Figure 3B) [37] and *C. elegans* (PDB accession no.: 1ZL9; Figure 3C) [38].

### Phylogenetic characterization of *Bsc*-GSTσ

**Figure 4:**
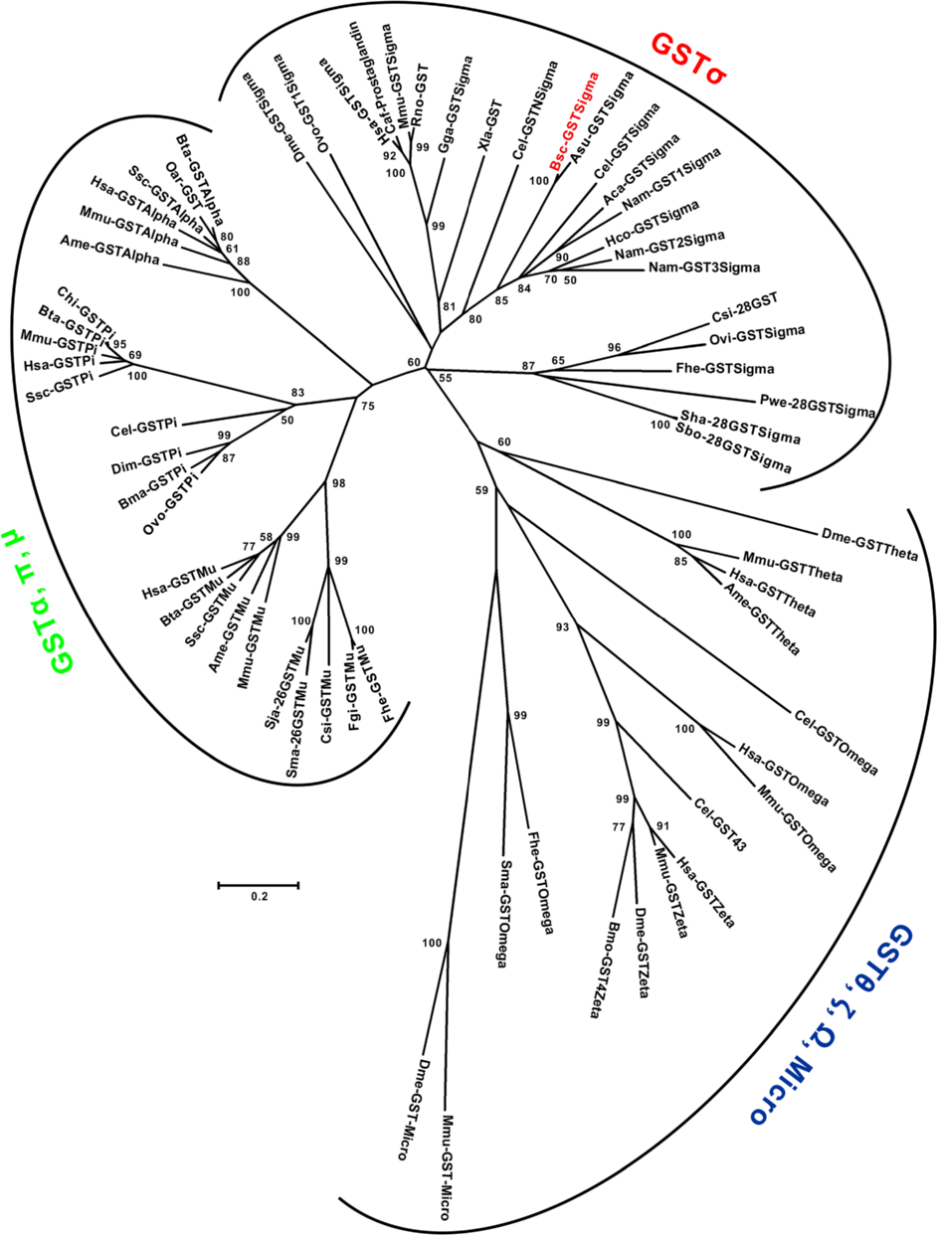
**Phylogenetic relationships of** ***B. schroederi Bsc*** **-GSTσ and other classes of GSTs.** An unrooted phylogenetic tree was inferred by neighbor-joining (NJ) analysis. Representative GSTs from eight classes: α, σ, Ω, θ, ξ, μ, π, and Micro. The tree was constructed from a multiple sequence alignment performed using ClustalW2 and plotted using MEGA 5.0. Based on the GTR + I + G model, 1,000 bootstrap replicates were run to calculate the percentage reliability for each node, and only values ≥50% are shown. The scale indicates an estimate of substitutions per site, using the optimized model setting. The protein sequences used in the tree are listed as follows, with their GenBank or SwissProt accession numbers: *Bos taurus* alpha GST (Bta-GSTAlpha), Q28035; *Ovis aries* GST (Oar-GST), Q9XS30; *Sus scrofa* alpha GST (Ssc-GSTAlpha), P51781; *Homo sapiens* alpha GST (Hsa-GSTAlpha), P08263; *Mus musculus* alpha GST (Mmu-GSTAlpha), Q6P8Q0; *Ailuropoda melanoleuca* alpha GST (Ame-GSTAlpha), XP_002923004; *Drosophila melanogaster* sigma GST (Dme-GSTSigma), P41043; *Onchocerca volvulus* sigma GST (Ovo-GSTSigma), P46434; *H. sapiens* sigma GST (Hsa-GSTSigma), O60760; *Canis familiaris* prostaglandin D synthase (Caf-Prostaglandin), NP_001186973; *M. musculus* sigma GST (Mmu-GSTSigma), Q9JHF7; *Rattus norvegicus* GST (Rno-GST),O35543; *Gallus gallus* sigma GST (Gga-GSTSigma), O73888; *Xenopus laevis* GST (Xla-GST), AAH53774.1; *C. elegans* sigma GST N (Cel-GSTNSigma), AAB65417; *B. schroederi* sigma GST (Bsc-GSTSigma), KM435257; *A. suum* sigma GST (Asu-GSTSigma), P46436; *C. elegans* sigma GST (Cel-GSTSigma), P91253; *A. caninum* sigma GST (Aca-GSTSigma), AAT37718; *N. americanus* sigma GST1 (Nam-GST1Sigma), ACX53261; *H. contortus* GST (Hco-GST), Q9NAW7; *N. americanus* sigma GST2 (Nam-GST2Sigma), ACX53262; *N. americanus* sigma GST3 (Nam-GST3Sigma), ACX53263; *Clonorchis sinensis* 28-kDa GST (Csi-28GST), O97096; *Opisthorchis viverrini* sigma GST (Ovi-GSTSigma), AAL23713; *Fasciola hepatica* sigma GST (Fhe-GSTSigma), ABI79450; *Paragonimus westermani* 28-kDa sigma GST (Pwe-28GSTSigma), AAB63382; *Schistosoma haematobium* 28-kDa sigma GST (Sha-28GSTSigma), AAA29892; *Schistosoma bovis* 28-kDa sigma GST (Sbo-28GSTSigma), AAA29893; *C. elegans* omega GST (Cel-GSTOmega), P34345; *H. sapiens* omega GST (Hsa-GSTOmega), P78417; *M. musculus* omega GST (Mmu-GSTOmega), O09131; *F. hepatica* omega GST (Fhe-GSTOmega), AFX98104; *Schistosoma mansoni* omega GST (Sma-GSTOmega), Q86LC0; *D. melanogaster* theta GST (Dme-GSTTheta), P20432; *M. musculus* theta GST (Mmu-GSTTheta), Q91X50; *H. sapiens* theta GST (Hsa-GSTTheta), P30711; *A. melanoleuca* theta GST (Ame-GSTTheta), XP_002925113; *H. sapiens* zeta GST (Hsa-GSTZeta),O43708; *M. musculus* zeta GST (Mmu-GSTZeta), Q9WVL0; *D. melanogaster* zeta GST (Dme-GSTZeta), Q9VHD3.1; *Bombyx mori* zeta GST4 (Bmo-GST4Zeta), ABC79691;*C. elegans* GST 43 (Cel-GST43), Q9N4H6; *H. sapiens* mu GST (Hsa-GSTMu), P09488; *B. taurus* mu GST (Bta-GSTMu), Q9N0V4; *S. scrofa* mu GST (Ssc-GSTMu), Q000H8; *A. melanoleuca* mu GST (Ame-GSTMu), XP_002919259; *M. musculus* mu GST (Mmu-GSTMu), P10649; *Schistosoma japonicum* 26-kDa mu GST (Sja-26GSTMu), P08515; *S. mansoni* 26-kDa mu GST (Sma-26GSTMu), P15964; *C. sinensis* mu GST (Csi-GSTMu), AAB46369; *F. gigantica* mu GST (Fgi-GSTMu), AAD23997; *F. hepatica* mu GST (Fhe-GSTMu), AHC02709; *Capra hircus* pi GST (Chi-GSTPi), Q9TTY8; *B. taurus* pi GST (Bta-GSTPi), P28801; *M. musculus* pi GST (Mmu-GSTPi), P19157; *H. sapiens* pi GST (Hsa-GSTPi), P09211; *S. scrofa* pi GST (Ssc-GSTPi), P80031; *C. elegans* pi GST (Cel-GSTPi), P10299; *Dirofilaria immitis* pi GST (Dim-GSTPi), P46426; *Brugia malayi* pi GST (Bma-GSTPi), CAA73325; *O. volvulus* pi GST (Ovo-GSTPi), P46427; *D. melanogaster* micro GST (Dme-GST-Micro), AAC98692; *M. musculus* micro GST (Mmu-GST-Micro), NP_064330.

To probe the evolutionary position of *Bsc*-GSTσ, the amino acid sequences of 64 GSTs included here were aligned and subjected to phylogenetic analysis (Figure 4). The constructed NJ tree clearly supported three major groups: Group 1 (GSTσ), Group 2 (GSTα + GSTπ + GSTμ), and Group 3 (GSTθ + GSTξ + GSTΩ + GST-Micro). Obviously, Group 3 maintained a greater genetic distance than that between the other two groups, with considerable statistical support (55%). Among Group 3 and particular Group 2, there were many small clusters formed by different GST classes (e.g., α, π, μ, θ, ξ, Ω, and Micro) with strong nodal supports (all bootstrap values ≥83% or =100%). For Group 1, the two helminth subgroups (nematodes and trematodes) formed distinct branches with strong supports (80% and 87%, respectively). In contrast with the invertebrate helminths, vertebrates formed another independent subgroup with a strong support (81%) within Group 1. Interestingly, the *Bsc*-GSTσ in the nematode-specific subgroup grouped with the GSTσ of *A. suum* (100%) and then with homologues of three strongylid worms (84%) and *C. elegans* (≥80%; see Figure 4).

### Expression, purification, and reactivity of r*Bsc*-GSTσ

**Figure 5:**
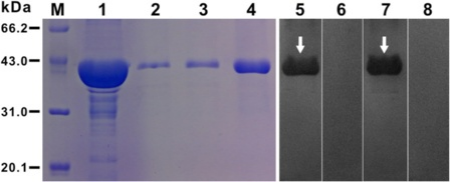
**SDS-PAGE and western blotting analysis of recombinant** ***Bsc*** **-GSTσ (r** ***Bsc*** **-GSTσ) protein.** M, molecular weight marker in kDa; lane 1, IPTG-induced *E. coli* BL21 (DE3) lysate; lanes 2–3, different concentrations of purified r*Bsc*-GSTσ after dialysis; lane 4, the refolded protein after treatment with the vacuum freeze-drying technique; lanes 5–8: refolded r*Bsc*-GSTσ probed with rabbit immune serum against *B. schroederi* (experimental group, lane 5), naïve rabbit serum (negative control, lane 6), anti-r*Bsc*-GSTσ mouse serum (positive control, lane 7), and pre-immune mouse serum (negative control, lane 8), respectively. The protein was stained with Coomassie Blue R250 in the gel (lanes 1–4), while the protein bound to serum samples was detected using NBT/BCIP in the immunoblotting (lanes 5–8). Two white arrows indicate the location of r*Bsc*-GSTσ-specific bands.

The cDNA encoding mature *Bsc*-GSTσ was successfully sub-cloned into the pET32a (+) expression vector (Invitrogen) and expressed in *E. coli* BL21 (DE3) cells as a single His 6-tagged fusion protein, with an expected size of ~42 kDa (lane 1, Figure 5). Due to an additional 20-kDa epitope tag fusion peptide, the molecular mass of r*Bsc*-GSTσ was ~22 kDa, similar to that predicted from its amino acid sequence. Peak expression levels of r*Bsc*-GSTσ occurred at 5 h induction with IPTG and occurred mostly in inclusion bodies. The r*Bsc*-GSTσ was purified using a single-step Ni-NTA affinity chromatography under denaturing conditions (containing 8 M urea). After refolding and concentration, the purity and yield (~6 mg/L) of r*Bsc*-GSTσ were accessed by SDS-PAGE (Figure 5, lanes 2–4). For western blotting analysis, a positive band of 42 kDa was observed when using both rabbit anti-*B. schroederi* serum (experimental group) and mouse anti-r*Bsc*-GSTσ serum (positive control), suggesting a strong reactivity and good antigenicity of this recombinant protein (Figure 5, lanes 5 and 7). No signal was present in the r*Bsc*-GSTσ incubated with native rabbit and mouse sera (lanes 6 and 8, Figure 5).

### Immunolocalization of native *Bsc*-GSTσ in adult female *B. schroederi*

**Figure 6:**
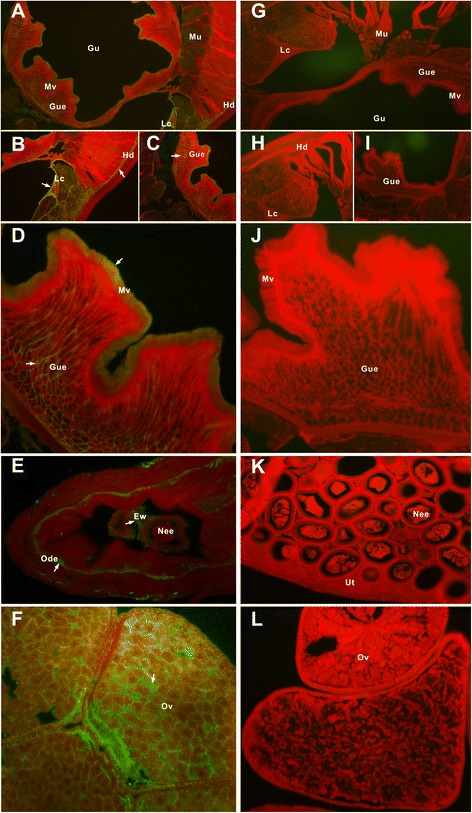
**Localization of endogenous** ***Bsc*** **-GSTσ in female adult** ***B. schroederi*** **using immunofluorescence.** Worms were fixed in paraformaldehyde and embedded in paraffin as described in Methods. The sections (5-μm thickness) were incubated with either mouse anti-r*Bsc*-GSTσ serum (1:100; panels **A-F**) or pre-immune serum at (1:100; panels **G-L**). The endogenous *Bsc*-GSTσ becomes visible with fluorescein isothiocyanate (FITC)-labeled goat anti-mouse IgG (1:100). White arrows indicate antibody-labeled regions. Different magnifications are used to highlight these regions and they include 10× **(A)**, 20× (**B**, **C**, **G**, and **I**), and 40× (**D-F**, **H**, and **J-L**). Abbreviations: Hd, hypodermis; Mu, muscle; Lc, lateral chord; Gu, gut; Gue, gut epithelium; Mv, microvilli; Ov, ovary; Ut, uterus; Od, oviduct; Ode, oviduct epithelium; Nee, non-embryonated eggs; Ew, egg wall.

The tissue distribution of the endogenous *Bsc*-GSTσ proteins was located by immunofluorescence assay using anti-r*Bsc*-GSTσ mouse serum and native mouse serum. Specific fluorescence was clearly observed in sections probed with anti-*Bsc*-GSTσ specific serum (Figure 6, panels A-F) but not in those probed with normal mouse serum (Figure 6, panels G-L). The results showed that endogenous *Bsc*-GSTσ proteins were mainly localized in several tissues or organs, including the hypodermis, lateral chords, gut epithelium, and gut microvilli of a female adult *B. schroederi* (panels A-D, Figure 6), which was consistent with its homologous protein in *A. suum* [36]. Interestingly, we detected strong fluorescence for *Bsc*-GSTσ in the ovaries, oviduct epithelium, and egg walls within oviducts, and a lack of fluorescence in muscles and eggs: this was in contrast to its counterparts in *A. suum* (panels E and F, Figure 6).

### Diagnostic potential of r*Bsc*-GSTσ protein

Given the strong antigenicity and reactivity of r*Bsc*-GSTσ, an ELISA-based serodiagnostic method was established. After screening combinations of various amount of antigens tested with various dilutions of positive polyclonal sera, 2 μg/mL of r*Bsc*-GSTσ antigen and 1:800 dilution of sera were deemed optimal for the full set of sample tests. The specific IgG antibodies in all serum samples of mice infected with *B. schroederi* or with *B. transfuga* and *A. suum* were determined (Table 1). Based on the negative cut-off of 0.194, a total of 34 serum samples from mice infected with *B. schroederi* were detected as positive, corresponding to a sensitivity of 79.1% (34/43). However, due to cross-reactivity with *B. transfuga*-positive mouse sera (N = 4) or *A. suum*-positive mouse sera (N = 5) and no reactions with normal mouse sera, the specificity of the ELISA using r*Bsc*-GSTσ antigen to detect *B. schroederi* was 82.0% (41/50). Nevertheless, there were statistical differences observed in the ELISA values between the *B. schroederi*-positive sera and the heterogeneous or control sera (*P* < 0.05; data not shown). No difference was noted between the heterogeneous and the control sera.

**Table 1:** **Detection of anti-** ***B. schroederi*** **IgG antibodies in serum samples of experimentally infected mice (N = sample size)**

**Sera of mice infected with parasites**	**N**	**r** ***Bsc*** **-GSTσ-ELISA**	
		**OD value (means ± SD)**	**No. of positive serum samples (%)**
*B. schroederi*	43	1.249 ± 0.0575	34 (79.1)
*B. transfuga*	15	0.396 ± 0.5223	4 (26.7)
*A. suum*	15	0.432 ± 0.5118	5 (33.3)
Control	20	0.113 ± 0.0270	0 (0)

## Discussion

Baylisascariasis, caused by the parasite *B. schroederi*, is today recognized as a significant cause of mortality of giant pandas, yet the diagnostic tools to detect *B. schroederi* infections in pandas are still lacking [3,39-42]. Numerous studies have highlighted the importance of parasite GSTs, particularly secretory GSTs, in parasite survival and host immune regulation, and thus, as potential candidates for the development of vaccines or diagnostic tools [15,16,20-22,37]. With this in mind, in this study we identified and characterized a novel, secretory sigma-class GST from *B. schroederi* (*Bsc*-GSTσ) and assessed its potential for serodiagnosis in an experimentally challenged mouse model.

The ongoing annotations of the genome and transcriptome of *B. schroederi* (within our research group) provide a comprehensive bioinformatics platform for probing the accurate complete reference sequence to design primers to amplify and express *Bsc*-GSTσ. By means of the His fusion-based pET32a (+) prokaryotic vector system, a ~42 kDa recombinant *Bsc*-GSTσ protein was generated here. Excluding the tag peptides, this ~22-kDa Mw was well within the range of 21–29 kDa reported for other GSTs [43] and agreed with the findings of a previous review that the average molecular mass of sigma GSTs in parasites is 22 kDa [14]. Further sequencing and structural analyses revealed that *Bsc*-GSTσ possessed the typical structural features of representatives of GSTs in the Sigma class: a coding domain for the GST_N_Sigma_like (PSSM: cd03039) and another for the GST_C_Sigma_like (PSSM: cd03192). Meanwhile, the signal peptide analysis implied that *Bsc*-GSTσ was a secretory protein. Previous studies have demonstrated that most parasite GSTs isolated to date, including some secretory GSTs, can be grouped independently of their host species into thirteen classes based on their sequence, structure, substrate specificity, and sensitivity to inhibitors (namely alpha, beta, kappa, delta, sigma, theta, mu, omega, pi, tau, zeta, psi, and epsilon) [13,44,45]. Combined, the results suggest that *Bsc*-GSTσ characterized here should be a secretory GST and is affiliated with the Sigma class.

Sequence alignment and phylogenetic analyses classified *Bsc*-GSTσ along with nematode-specific GSTσs, and it together with homologues of other nematodes and trematodes described to date formed a unique, major invertebrate subgroup and were distinct from those of the vertebrates in the Sigma class GSTs (Figure 4), which was consistent with a recently published report [20]. Within the nematode-specific GSTσs, eight homologous proteins derived form six parasite species, including *B. schroederi*, were further subjected to pair comparisons on primary, second, and tertiary structure levels. Although *Bsc*-GSTσ was most similar to *A. suum* GSTσ at the amino acid level, the N-terminal seven GSH-binding moieties responsible for the G-site were highly conserved (100% identity) across all the nematode species included here. Similar conservations were also indicated by their higher (secondary or 3D) structures where a classical GSTσ βαβαββα motif was present in the N-terminal domain (see Figure 2) [44]. In contrast with G-sit, the C-terminal substrate binding H-site seemed to be greatly variable. Studies confirm that the amino acid identity in the H-site of GSTs is usually very low, even between intra-class members (<35%) [46]. This provides the opportunity to screen the region-specific sites for immune responses or to design drugs to inhibit the parasite enzyme only. Furthermore, the distinction of *Bsc*-GSTσ from its counterpart in giant pandas further enhances its potential as a diagnostic or therapeutic target due to impossible autoimmune responses caused by cross-reactivity. Under this context, we explored the antigenicity and reactivity of *Bsc*-GSTσ using its recombinant form. The results of our immunoblotting analysis showed that r*Bsc*-GSTσ was strongly recognized by rabbit anti-*B. schroederi* serum and mouse anti-r*Bsc*-GSTσ serum and became visible at 42 kDa. These findings confirm the strong immunogenicity of this compound and its potential as a diagnostic tool or candidate target for vaccine development. Two recent studies have successfully used recombinant GSTσ antigens in vaccines against human hookworm *N. americanus* (e.g., r*Na*-GST-1) [47,48] and liver fluke *Fasciola hepatica* (e.g., rFhGST-S1) [20]. This work suggests that immunoprotective assays to determine the vaccine potential of this recombinant protein in *B. schroederi* are warranted. On the other hand, we characterized *Bsc*-GSTσ as a secretory antigen of *B. schroederi*. Previous studies have demonstrated the potential of parasite-specific E/S antigens in diagnostics [12,49-53], thus we thought it prudent to assess the potential of this compound in the serodiagnosis of *B. schroederi* infections in giant pandas. *B. schroederi* infections in wild pandas are not associated with specific clinical symptoms, and the infection can only be confirmed until worms are expelled or eggs are shed in the feces [5]. The development of a serodiagnostic tool will enhance existing diagnostic methods and improve treatment options, especially during the larval migrating stages (L2-L4). After optimizing conditions, a r*Bsc*-GSTσ-based ELISA was established in the present study. Our results revealed that r*Bsc*-GSTσ could clearly detect the *B. schroederi-*specific IgG antibodies in experimental mouse sera, with a sensitivity of 79.1% and a specificity of 82.0%, although cross-reactions were observed in several *B. transfuga*- or *A. suum*-positive mouse sera. The presence of cross-reactions may be associated with a similar distribution of epitopes in the GSTσ homologues of the two ascaridoids that are closely related to *B. schroederi*. A similar phenomenon was also described between other parasitic nematodes (between *Toxocara canis* and *A. suum* and *Toxascaris leonina* [54], between *Anisakis simplex* and *A. suum* and *Anisakis physeteris* [55], and between *Baylisascaris procyonis* and *Toxocara* spp. [56]). The sensitivity of the assay was not high, and is likely a consequence of using a recombinant antigen for a serodiagnostic assay. Recombinant antigens are often produced as single proteins and lack the post-translational chemical or structural modifications present in its native counterpart, which can weaken their sensitivity. Interestingly, it has been shown that combining recombinant antigens can improve the sensitivities of recombinant antigens in the serodiagnosis of parasitic infections [56,57]. Nevertheless, it is important to note that the geographic range of giant pandas does not overlap with that of bears (the hosts for *B. transfuga*) or swine (the hosts for *A. suum*). Hence, the chance of cross-reactivity in the wild is low and the serodiagnostic ELISA developed here would be able to detect *B. schroederi* in pandas with a respectable sensitivity.

Finally, our localization analysis showed that native *Bsc*-GSTσ was present in the intestine, reproductive tissues, and body hypodermis of female adult *B. schroederi*. Within the intestine a strong immunofluorescent signal for *Bsc*-GSTσ was observed in the brush border of the microvilli, as has been reported for its homologue AsGST1 in *A. suum* [37]. Surprisingly, this intestinal location was distinct from its homologue OvGST1 in the filaria *O. volvulus*: the endogenous OvGST1 was distributed in the hypodermis [58]. This discrepancy may be associated with species-specific differences amongst nematodes. With respect to filarial nematodes, they are being considered to become specialized and adopt a blood-based or lymph-based nutrition parasitism, especially their intestines [59]. As compensation for the degeneration or loss of intestinal function, the presence of OvGST1 in the hypodermis of *O. volvulus* is probably responsible for the metabolism of extrinsic materials and the excretion or secretion of molecules from the parasite, similar to that which occurs in the intestines [14,15]. This finding suggests that *Bsc*-GSTσ in *B. schroederi* and AsGST1 in *A. suum* may both be involved in metabolism and detoxification as well as nutrition during the development of ascaridoids. In addition, we also observed strong fluorescent signals of *Bsc*-GSTσ in the ovaries, oviduct epithelium and egg walls, but not in the muscles or ooplasm of *B. schroederi*. This contrasts to the results reported for their counterparts in *A. suum* [37]. Whether the expression of this gene in these tissues is also nematode species-specific is not known, but the expression of GSTs in trematode parasites has been reported to be tissue-specific and developmentally regulated. For example, Sm26GST and Sm28GST in *Schistosoma mansoni* were located in the parenchymal cells of adults but were absent from the musculature, gut and reproductive tissues [60,61]. In *F. hepatica*, a more recent study showed that FhGST-S1 was present in the parasite tegument, parenchyma, musculature and eggs, and was also found in the excretory/secretory fraction of adults. Thus, the authors inferred that the FhGST-S1 of the adult fluke may play a role in parasite development and the interaction between host and parasite [20]. Perhaps, with the advent of readily available genomic tools, the combination of high-throughput genome sequencing and mass spectrometry techniques for exploring pan-nematode proteomics will provide a comprehensive platform for an in-depth structure and function analyses of these GSTσs. This should contribute to a better understanding of the biological roles of nematode-specific GSTσs.

## Conclusions

The full-length cDNA encoding a novel Sigma class glutathione S-transferases (*Bsc*-GSTσ) from *B. schroederi* was identified by screening the genome and trancriptome datasets and subsequently cloned and expressed. We determined some of the structural characteristics and tissue-specific distributions of this compound, providing insights into the biological functions of this protein. Furthermore, we showed that r*Bsc*-GSTσ has strong immunogenicity and we confirmed via a serodiagnostic assay that r*Bsc*-GSTσ is a suitable diagnostic antigen and could be used to develop an ELISA-based serological test for the diagnosis, seroepidemiology, and serosurveillance of *B. schroederi* in giant pandas.
